# Religiosity and quality of life of individuals with Alzheimer’s disease and of caregivers: Relationship with clinical aspects

**DOI:** 10.1590/1980-57642020dn14-010011

**Published:** 2020

**Authors:** Gloria Maria A.S. Tedrus, Lineu Correa Fonseca, Julio Cesar Bredas Ciancaglio, Gabriela Scartezini Mônico, Carolina Zamperi

**Affiliations:** 1 Postgraduate Program in Health Sciences, Pontifícia Universidade Católica de Campinas, Campinas SP, Brazil.; 2 Undergraduate student. School of Medicine, Pontifícia Universidade Católica de Campinas, Campinas SP, Brazil.

**Keywords:** Alzheimer’s disease, quality of life, religiosity, family caregiver, doença de Alzheimer, qualidade de vida, religiosidade, familiar cuidador

## Abstract

There are few studies on the religiosity of people with Alzheimer’s disease (PwAD) and caregivers, relation with quality of life (QoL) and clinical aspects.

**Objective:**

To assess the religiosity and QoL of 39 PwAD and their caregivers; to compare perceived QoL and religiosity of the PwAD with those of their caregivers; to associate QoL and religiosity with the presence of neuropsychiatric symptoms, and depression with cognitive performance of PwAD.

**Results:**

Organizational religiosity was greater in caregivers. The AD patients had poorer perceived QoL than their caregivers. Caregiver religiosity correlated with that of the AD patients. Higher intrinsic religiosity was associated with lower occurrence of neuropsychiatric symptoms. Better caregiver QoL correlated with cognitive performance. Lower occurrence of depression correlated with better QoL of the caregivers and AD patients.

**Conclusion:**

The religiosity of caregivers was correlated with that of the AD patients. Better QoL and lower religiosity were observed in caregivers when compared with the AD patients. Caregiver religiosity and QoL were associated with neuropsychiatric and cognitive aspects and depression.

## RESUMO

São poucos os estudos da religiosidade de indivíduos com doença de Alzheimer (AD) e de cuidadores e as relações com a qualidade de vida (QV) e aspectos clinicos.

**Objetivo:**

Relacionar os dados do *Duke University Religion Index* e da *Quality of life in Alzheimer’s disease scale* de 39 idosos (AD: leve ou moderada) e do cuidador com aspectos clínicos, cognitivos e comportamentais.

**Resultados:**

religiosidade organizacional é maior nos cuidadores. Idosos com AD percebem pior QV do que o cuidador. Religiosidade do cuidador correlacionou-se com a dos idosos. Maior religiosidade intrínseca relacionou-se com menor ocorrência de sintomas neuropsiquiátricas. Melhor QV dos cuidadores correlacionou-se com desempenho cognitivo. Menor ocorrência de depressão correlacionou-se a melhor QV dos cuidadores e dos idosos.

**Conclusão:**

A religiosidade dos cuidadores relaciona-se com a dos idosos. Melhor QV e menor religiosidade foi observada nos cuidadores quando comparadas a dos idosos. Religiosidade e QV dos cuidadores associam-se com aspectos neuropsiquiátricos, cognitivos e com a ocorrência de depressão.

Studies suggest a relationship between spirituality, religiosity and health in several contexts and highlight the positive aspects in quality of life (QoL) and general well-being.^1^^,^ 2 However, this relationship is complex and bidirectional and, despite increased scientific interest, several mechanisms involved in this relationship are still unknown.^1^^,^ 3

Spirituality and religiosity are cultural factors that give meaning to life and may help coping with situations of stress, particularly in chronic diseases, and also promote a positive outcome in health indices.^3^ Studies have suggested that increased religiosity during negative life events, such as during illnesses, may lead individuals to use positive or negative patterns of religious coping strategies to deal with a disease in situations of stress.^4^

Few topics in medicine have given rise to more disagreement than the study of the relationship between religion and health. It is well known that patient opinions on the relationship between healthiness and health differ according to their religion and different meanings and values attributed to them, to whether they are a theist or an atheist, and the characteristics of their health problems.^1^^,^ 5 ^,^ 6 Other controversial aspects inherent to studying this topic include understanding the wide range of terms for religiosity and spirituality, the different measurement instruments, populations studied and data interpretation.^6^^,^ 7

However, in Alzheimer’s disease (AD) there are still knowledge gaps regarding beliefs and religious practices when dealing with clinical aspects of the disease, practices which might improve the QoL of people with Alzheimer’s disease (PwAD) and their caregivers. Studies suggest that spirituality and religiosity may slow cognitive decline in AD, help coping with the disease, and promote better QoL.^5^^,^ 6 However, authors have suggested that these results should be interpreted with caution and highlight the need for further consistent research.^7^

The hypothesis of this study is that religiosity intensity and perceived QoL of caregivers are related to disease severity, presence of depression and neuropsychiatric symptoms in PwAD.

Thus, the aim of this study was to assess aspects of religiosity and QoL of PwAD and their caregivers, to compare perceived QoL and religiosity of the PwAD with those of their caregivers, and to correlate QoL and religiosity with the presence of neuropsychiatric symptoms, and depression with cognitive performance of PwAD.

## METHODS

### Subjects

The sample consisted of 39 patients over the age of 65 years with a clinical diagnosis of probable AD. The patients were seen at the neurology outpatient clinic of the Hospital of the Pontifical Catholic University of Campinas, Campinas, São Paulo state, Brazil.

The inclusion criteria for the PwAD were as follows: diagnosis of probable AD according to the criteria of the Diagnostic and Statistical Manual of Mental Disorders (DSM-IV)^8^ in accordance with the recommendations of the National Consensus criteria for the diagnosis of probable AD.^9^^,^ 10 Dementia severity was classified on the Clinical Dementia Rating^11^ as mild in 24 cases and moderate in 15 cases. All patients lived at home with their families. All patients were on cholinesterase inhibitors.

Exclusion criteria included the presence of other serious diseases that caused reduction in life expectancy and inability to complete the cognitive assessment instruments.

The family caregivers who accompanied the elderly patients to medical appointments were included in the study. The caregivers and PwAD were invited to participate and were fully informed about the study. All participants and/or persons responsible for the PwAD signed an informed consent form. The study was approved by the Human Research Ethics Committee of PUC-Campinas.

### Procedures

The following instruments were used to assess the PwAD:

- Neurological and cognitive assessments: consisted of an objective interview with patient and informant, analysis of routine laboratory exams and application of the following cognitive batteries: the Mini-Mental State Examination (MMSE),^12^^,^ 13 Category fluency test (animals in one minute), Clock-Drawing Test; standard neuropsychological battery of the Consortium to Establish a Registry for Alzheimer’s Disease (CERAD).^14^ The Clinical Dementia Rating^11^ was applied for staging of the severity of dementia.
- Rating Scale for Depression (RSD):^15^ this is a 24-item self-assessment depression scale to quantify the presence and severity of symptoms over the past week. The total score was used. The higher the total score, the more severe the depression.
- Duke University Religion Index – Brazilian Portuguese version (PDUREL):^16^ this is a five-item measure of religious involvement, which yields three subscales: a) organizational religious behavior (OR), b) nonorganizational religious behavior (NOR), and c) intrinsic religious motivation (IR). Response options are scored on a 5- or 6-point Likert scale. Lower scores indicate lower religiosity.

The following instruments were used to assess the caregivers:

- Collection of sociodemographic data: age, religion, marital status, level of education and degree of kinship with the elderly AD patient;
- Neuropsychiatric Inventory (NPI):^17^ this is a 10-item questionnaire that assesses the frequency and severity of neuropsychiatric symptoms in AD patients.
- Quality of life in Alzheimer’s Disease Scale – caregiver version (CPQdV-DA). and AD patient version (according to caregiver) (CQdV-DA):^18^ the instrument consists of 13 items scored from 1-4 points (1 = bad; 4 = excellent). The total score ranges from 13 to 52 and higher values indicate better perceived QoL. The scale was validated and adapted for the Brazilian population.^19^
- Disability Assessment for Dementia (DAD):^20^ this comprises an instrument that measures the functional abilities in individuals with cognitive deficits during daily activities without the help of a caregiver. The total score is obtained by adding up the items and converting them into a percentage. Higher scores indicate less disability in daily activities. The version adapted for the Brazilian culture was used.^21^

### Statistical analysis 

Quantitative variables were expressed as means and standard deviations (SD), while qualitative variables were expressed as frequency and percentage values (%). The two-tailed Student’s *t*-test was applied to compare continuous variables, while Fisher’s exact or Pearson’s Chi-square tests were used to compare qualitative data and frequencies of occurrence. The Spearman Correlation coefficient was used to measure the degree of associations among the quantitative variables.

The data were treated using the software IBM SPSS Statistics, version 22. A significance level of *p*<0.05 was used for the statistical tests.

## RESULTS

### Sociodemographic and clinical aspects

The sociodemographic and clinical aspects of the PwAD and caregivers are shown in Table 1. PwAD were older and had fewer years of formal education when compared with the caregivers. There was a predominance of women among caregivers. Regarding religious belief, 60.5% were catholic.

**Table 1:** Sociodemographic and cognitive aspects, and scores on the Rating Scale for Depression, Disability Assessment for Dementia, Neuropsychiatric Inventory, Quality of life in Alzheimer's Disease Scale - caregiver version, Quality of life in Alzheimer's Disease Scale - AD patients, and Duke University Religion Index of AD patients and their caregivers.

		AD patients (n = 39)	Caregiver (n = 39)	p
Age (years, mean ± SD)		78.9 (± 7.2)	52.4 (± 14.0)	<0.001^a^ *
Level of education (years, mean ± SD)		3.5 (± 2.5)	8.6 (± 4.3)	<0.001^a^ *
Sex (male/female)		15/ 24	7/ 32	0.044^b^ *
Cognitive aspects	MMSE (mean ± SD)	16.4 (± 5.3)		
	VF animals (mean ± SD)	7.3 (± 3.5)		
	Word list memory (Boston naming) (mean ± SD)	9.2 (± 4.0)		
	Constructional praxis (mean ± SD)	5.8 (± 3.4)		
	Praxis recall (mean ± SD)	0.5 (± 1.2)		
RSD (mean ± SD)		11.5 (± 6.0)		
DAD	Basic activities (%)	84.1 (± 26.4)		
	Instrumental activities (%)	40.3 (± 30.6)		
NPI	Severity	8.5 (± 5.3)		
	Frequency	9.7 (± 6.5)		
Quality of life-AD (mean ± SD)		30.5 (± 6.4)	34.1 (± 6.4)	0.024^c^ *
PDUREL	OR (mean ± SD)	3.52 (± 1.6)	2.89 (± 1.6)	0.007^c^ *
	NOR (mean ± SD)	2.26 (± 1.35)	2.32 (± 1.35)	0.050^c^
	IR (mean ± SD)	4.43 (± 2.0)	4.3 (± 2.13)	0.522^c^

AD: Alzheimer's disease; MMSE: Mini-Mental State Examination; VF animals: category fluency test; RSD: Rating Scale for Depression; DAD: Disability assessment for dementia; NPI: neuropsychiatric inventory; PDUREL: Duke University Religion Index - Brazilian Portuguese version; OR: organizational religious behavior; NOR: nonorganizational religious behavior; IR: intrinsic religious motivation;

a t-test

b Chi-square test;

c paired t-test.

* p<0.05.

In the classification of dementia severity, a slight predominance of the moderate stage of the disease was observed (38.4%) on the CDR. The cognitive assessment data for the PwAD were compatible with the criteria for the diagnosis of dementia and classification of severity.

There was considerable impairment in instrumental activities and relative preservation of basic activities on the DAD (Table 1).

### PDUREL, QoL: PwAD, caregivers

Caregivers had better perceived QoL and higher organizational religious behavior (OR) when compared with the PwAD (Table 1).

There were significant correlations for the dimensions of the PDUREL between caregivers and PwAD (Table 2).

**Table 2:** Correlation between the Duke University Religion Index of caregiver and scores on the Disability Assessment for Dementia, Rating Scale for Depression, Neuropsychiatric Inventory, Quality of life in Alzheimer's Disease Scale - caregiver version - and Quality of life in Alzheimer's Disease Scale - AD patients.

	PDUREL							
	OR			NOR			IR	
	Correlation	p		Correlation	p		Correlation	p
DAD: basic activities	0.018	0.912		-0.004	0.978		-0.107	0.524
DAD: instrumental activities	0.232	0.150		0.191	0.238		0.162	0.331
NPI: severity	0.205	0.203		0.188	0.247		0.468	0.003*
NPI: frequency	0.173	0.286		0.169	0.297		0.425	0.008*
RSD	-0.004	0.981		0.162	0.326		0.212	0.207
OR (PDUREL)	0.606	<0.000*		0.340	0.046*		0.174	0.332
NOR (PDUREL)	0.346	0.041*		0.418	0.012*		0.546	0.001*
IR (PDUREL)	0.419	0.015*		0.125	0.487		0.094	0.602
CPQdV-DA	0.165	0.323		-0.144	0.389		-0.133	0.425
CQdV-DA	0.129	0.474		-0.056	0.755		-0.093	0.605

DAD: Disability assessment for dementia; RSD: Rating Scale for Depression; NPI: neuropsychiatric inventory; PDUREL: Duke University Religion Index - Brazilian Portuguese version; OR: organizational religious behavior; NOR: nonorganizational religious behavior; IR: intrinsic religious motivation; CPQdV-DA: Quality of life in Alzheimer's Disease Scale - caregiver version; CQdV-DA: Quality of life in Alzheimer's Disease Scale - AD patients. Spearman correlation coefficient.

* p<0.05.

A significant correlation was observed between caregiver IR and the severity and frequency of neuropsychiatric symptoms on the NPI (Table 2).

Better perceived QoL of the PwAD and caregivers was associated with lower scores on the RSD (Table 3).

**Table 3:** Correlation between Quality of life in Alzheimer's Disease Scale - caregiver version, the Quality of life in Alzheimer's Disease Scale - AD patients and sociodemographic data, cognitive data and scores on the Rating Scale for Depression, Disability Assessment for Dementia and Neuropsychiatric Inventory.

	CQdV-DA (n=39)			CPQdV-DA (n=39)	
	Correlation	p		Correlation	p
Age	0.149	0.371		0.232	0.156
Level of education	-0.086	0.607		0.137	0.137
MMSE	-0.186	0.263		0.150	0.362
VF animals	0.051	0.759		0.051	0.03*
RSD	-0.456	0.004*		-0.467	0.003*
DAD: basic activities	0.060	0.721		0.040	0.810
DAD: instrumental activities	-0.159	0.339		0.021	0.897
NPI: severity	0.116	0.489		-0.207	0.206
NPI: frequency	0.245	0.139		-0.096	0.563
CQdV-DA				0.375	0.024*

AD: Alzheimer's disease; MMSE: Mini-Mental State Examination; VF animals: category fluency test; RSD: Rating Scale for Depression; DAD: Disability assessment for dementia; NPI: neuropsychiatric inventory; CPQdV-DA: Quality of life in Alzheimer's Disease Scale - caregiver version; CQdV-DA: Quality of life in Alzheimer's Disease Scale - AD patients. Spearman correlation coefficient.

* p<0.05.

There was a correlation between the QoL of caregivers and QoL of PwAD. Better perceived QoL of caregivers was associated with better performance on the category fluency test (Table 3).

There was no significant correlation of QoL (PwAD and caregivers) with age, level of education or scores on the MMSE, DAD and NPI (Table 3).

## DISCUSSION

This study assessed aspects of religiosity and QoL of 39 consecutive elderly patients diagnosed with AD (mild and moderate) and their caregivers. We found that religiosity and QoL of PwAD and caregivers was correlated with clinical aspects and presence of neuropsychiatric symptoms. These data suggest that the caregivers’ religiosity is a coping and adaptation strategy in situations of stress when dealing with the health-disease-care process.

The sociodemographic characteristics of the caregivers were similar to those described in the literature, in which there is a predominance of women, preferably wives – or affectively close daughters – who reported that they perform this activity due to cultural norms of caregiving in the family environment.^22^

The data obtained for religious affiliation in the present study were similar to those found in data from the 2010 demographic census carried out by the Brazilian Geography and Statistics Institute.^23^

### Religiosity: clinical and behavioral aspects

It is well known that religiosity/spirituality could be a factor for improving perceived QoL, as it provides comfort and reference in the pursuit of well-being, health, dignity, significance, and the construction of meanings for human life.^5^^,^ 24 ^,^ 25

A correlation between the intensity of OR, IR and NOR among the PwAD and caregivers was observed, which suggest similar conditions of religious experience in the family. The caregivers of more religious PwAD participate more frequently in religious activities and pray more at home. However, religiosity and spirituality are unique personal experiences of each individual and a sacred realm of human experience.^6^^,^ 26

Greater severity and frequency of neuropsychiatric symptoms in PwAD are associated with lower IR of the caregiver. The presence of non-cognitive symptoms in AD can influence behavioral changes by making the caregiver increasingly more important and responsible in the caregiving process. Thus, such symptoms may influence the caregivers by increasing their emotional and physical burden, which may lead to negative patterns of religious coping strategies when dealing with the worsening of the disease.^4^^-^ 6

Although studies suggest that religious involvement is associated with favorable trajectories of cognitive function, the explanations for this relationship are unclear.^5^^,^ 6 However, identifying and supporting religious coping strategies, promoting the use of spiritual resources that provide a meaning to life and reduce suffering, and the social support offered by religious groups can help PwAD and caregivers to better deal with the disease.

### QoL: clinical and behavioral aspects

Caregivers had better perceived QoL than the PwAD. Evidence suggests that patients with AD are not able to respond accurately and consistently to QoL issues, which may influence how PwAD and caregivers perceive QoL.^27^^-^ 29 Alzheimer’s disease not only affects elderly patients by causing suffering, loneliness and vulnerability, but also impacts caregivers and can impair the QoL of both PwAD and caregivers.^30^^-^ 33

It is known that different views on the same problem can change the way the disease, and consequently health care, is dealt with. The assessment of the QoL of the caregiver is important because the data collected and analyzed can help provide support to caregivers when caring for themselves and the PwAD.

The perceived QoL of PwAD and caregivers was negatively affected by depression, a relationship also found in other studies.^31^ Studies suggest that the correlation between lower occurrence of depression in PwAD and caregivers’ better perceived QoL could be related to a vicious cycle in which the presence of depressive symptoms in caregivers may worsen the QoL of PwAD or the depressed PwAD may negatively affect the QoL of caregivers.^27^^,^ 29 ^-^ 31

The occurrence of depression in AD directly affects daily activities and sleep of the elderly patients, which can increase stress perceived by the caregiver and require training and coping skills.^31^^,^ 32 Cognitive and sociodemographic aspects and neuropsychiatric symptoms in the elderly with dementia are associated with QoL and burden experienced by the caregiver.^30^

Caregivers’ better perceived QoL was related to improved performance on the category fluency test, suggesting a complex interrelationship between the cognitive impairment of PwAD and perceived QoL of the caregiver. Changes in verbal fluency may occur in the early stages of the progressive impairment of AD clinical condition, which consequently demands more from caregivers as care becomes a much more complex task.^27^^,^ 31 In a longitudinal study that assessed QoL and religiosity in AD, no relationship was found between QoL and disease progression. However, slower progression of cognitive impairment was associated with higher levels of spirituality and private religious practices.^32^

In summary, the study results suggest a relationship between caregiver and patient religiosity. The occurrence of depression and the presence of neuropsychiatric symptoms were associated with religiosity and QoL in AD.

### Limitations of the study

There are a few limitations to the results of the present study. Although standardized scientifically validated instruments were used, there are certain limitations imposed by the small number of cases from a single institution. Our clinic is based within a university hospital, but is not a tertiary center. Since the study was conducted at a single healthcare facility, cross-cultural comparison was not possible.
